# Comparison of the Knowledge, Attitudes, and Practices Regarding Silver Diamine Fluoride (SDF) between Japanese Dental Students with Experience Using SDF and Those with No Experience Using SDF: A Cross-Sectional Study

**DOI:** 10.3390/dj11120282

**Published:** 2023-12-05

**Authors:** Yuko Fujita

**Affiliations:** Division of Developmental Stomatognathic Function Science, Department of Health Promotion, Kyushu Dental University, Kitakyushu City 803-8580, Japan; y-fujita@kyu-dent.ac.jp

**Keywords:** silver diamine fluoride, dental caries, children, dental students, dental education

## Abstract

Background: The aim of this study was to evaluate the differences in the knowledge and attitude regarding silver diamine fluoride (SDF) between two groups, differentiated by whether they had experience in SDF use, of dental students and clinical trainee dentists in Japan. Methods: A survey was designed consisting of three dental classes (fourth, fifth, and sixth years) and clinical trainees at Kyushu Dental University. A survey was designed consisting of 32 questions about the knowledge, attitudes, esthetic acceptability, and potential barriers regarding the use of SDF. Results: A total of 286 surveys (response rate of 85.4%) were collected. Among all respondents, 21.7% had experience with SDF use in their clinical practice. Regarding the knowledge score for SDF (0 to 12 points), in the respondents with no experience of using SDF, the mean score was 3.06, and that of respondents with experience of using SDF was 1.66, which was a significant difference (*p* < 0.001). The mean esthetic acceptability score for SDF use (−8 to 8 points) of the clinical trainees was −1.00 and that of the fourth-year students was 0.74, which was a significant difference (*p* < 0.05). Conclusions: the results indicate that dental students and clinical trainees need to increase their clinical experience with SDF.

## 1. Introduction

Silver diamine fluoride (SDF) was developed by Professor Yamaga of Osaka University in Japan in the 1960s [1]. The inhibition technique was based on the disinfecting properties of silver and the remineralizing effect of fluoride. SDF has been reported to have significant antibacterial activity, an inhibition of demineralization and collagen degradation, and its safety has been proven [2]. Around the 1970s, at a time when childhood cavities were rampant and there was a shortage of dentists in Japan, SDF was frequently used in local dental clinics for the management of dental caries in children [1]. However, it has become less used in Japan since the 1990s, when the materials and techniques for restoration made remarkable advances [3].

In 2014, the US Food and Drug Administration (FDA) cleared the first SDF product in the United States for use as a “device” to treat hypersensitivity, and the product has a similar control pathway to the fluoride varnish’s clearance [4]. In 2016, the FDA granted the designation of breakthrough therapy to Advantage Arrest 38% SDF for arresting dental caries in both children and adults [4]. The American Academy of Pediatric Dentistry supports the use of 38% SDF in primary teeth to control cavitated lesions as part of a comprehensive caries management plan [5]. Since the approval of SDF by the FDA, it is now available for use in dental clinics in European countries. In 2020, the British Society of Paediatric Dentistry (IAPD) published its support for the use of SDF to treat caries. In 2021, the World Health Organization (WHO) included SDF in the essential health system medicine that meets the most important needs of adults and children [6]. It is very interesting that a situation opposite to that in Japan is occurring in Europe and America.

Despite various advantages, the most obvious disadvantage of SDF is the side effect of the permanent black staining of carious lesions [7]. Surveys of dental practitioners in the Netherlands and the US reported that the main barrier to the use of SDF is a lack of knowledge, followed by dental professionals’ concern regarding the parental acceptance of the black staining [8,9].

Currently, dental schools in Japan educate their students about SDF, but there is no information on how well they educate their students or how well dental students understand SDF. For the aforementioned reasons, it is possible that the content of SDF education for Japanese dental students has been reduced over the past few decades. However, despite advances in the treatment of dental caries over time, there has been a major problem with the dental caries’ prevalence among children in Japan in recent years. It has been reported that the dental caries’ prevalence in 5- to 9-year-old children has decreased considerably. However, the latest data from 2022 show that 17.9% of 5-year-old children, 35.3% of 7-year-old children, and 41.2% of 10-year-old children had experienced dental caries [10]. These results are similar to those reported in European countries with high socio-economic inequality [11,12]. 

Therefore, in order to thoroughly manage childhood caries, we need to reconsider the use of SDF, including its indications. This survey of dental students and clinical trainee dentists about their knowledge, attitude, and practices regarding SDF use in pediatric patients will provide insight into their perceptions on this topic and whether further education or training is needed to improve their attitudes towards its use.

The primary aim of this study was to assess the knowledge, attitudes, and practices regarding SDF among dental students and clinical trainee dentists in Japan and to explore the differences between the two groups, differentiated according to whether they had experience with SDF. We hypothesized that in the students and clinical trainee dentists with no experience of using SDF, the knowledge level of SDF would be lower, and their attitude toward its use would be more passive than that of the students and clinical trainees with experience using SDF.

## 2. Materials and Methods

This study was approved by the Human Investigations Committee of Kyushu Dental University (Kitakyushu, Fukuoka, Japan; Approval Number 23–21) in 2023, and all subjects provided written informed consent prior to participation.

### 2.1. Study Subjects

An a priori power analysis was conducted with the program package G*Power software (Power for windows version 3.1.9.4, available from the Heinrich-Heine-Universität Düsseldorf’s website) to calculate the sample size with an effect size 0.05, a power of 0.90, and a type I error probability for the null hypothesis of 0.05, in the linear regression model. We would need to survey 213 respondents to be able to reject the null hypothesis. The survey was completed by 247 dental students (120 women and 127 men) in three dental classes (fourth, fifth, and sixth years) and 39 clinical trainee dentists (16 women and 23 men) at Kyushu Dental University during September 2023. Inclusion criteria were fourth, fifth, and sixth year students enrolled in the Department of Dentistry, Faculty of Dentistry, Kyushu Dental University, and clinical trainees working at Kyushu Dental University Hospital. Subjects on leave of absence and subjects who could not consent to this survey were excluded. The lecture course about SDF in children takes place in April of the fourth year in the curriculum at this university. In the fifth and sixth years, students undertake clinical clerkship at the university hospital. Clinical trainee dentists are those who have passed the national exam and have less than one year of clinical practice at the university hospital.

### 2.2. Questionnaire

The author modified the questionnaires used in two previous studies that evaluated the perceptions of pediatric dentists or graduating dental students regarding dental treatment using SDF and the education, knowledge, attitudes, and professional behavior of dentists regarding the use of SDF [9,13]. A survey was designed consisting of 32 questions: 5 questions regarding the respondents’ characteristics, namely, sex, age, academic year, education about SDF, and clinical experience with SDF; 10 questions aiming to assess self-perceived general SDF knowledge (by using multiple-choice questions, where the possible answers were “yes”, “no”, or “I don’t know”), with correct answers from these 10 questions being summed to create a “Self-perceived general SDF knowledge score” index; 3 questions aiming to assess the subjects’ attitudes toward the general indications of SDF (by scoring statements based on the practitioner’s level of agreement using a 5-point Likert scale), with the scores from these 3 questions being summed to create an “Attitudes score on general indications to use SDF” index; 5 questions aiming to assess the subjects’ knowledge on the specific indications and practice of SDF (by scoring statements based on the practitioner’s level of agreement using a 5-point Likert scale), with the scores from these 5 questions being summed to create a “Knowledge score on practice of SDF” index; 4 questions aiming to investigate the subjects’ perceptions regarding the esthetic acceptability of SDF treatment (by scoring statements based on the practitioner’s level of agreement using a 5-point Likert scale), with the scores from these 4 questions being summed to create an “Esthetic acceptability score on SDF use” index; and 5 questions aiming to investigate the subjects’ perceptions of the potential barriers to the use of SDF (by using two-choice questions, where the possible answers were “agree” or “disagree”), with the scores from these 5 questions being summed to create a “Potential barriers score on SDF use” index. The components and order of the questionnaires filled out by respondents were as shown in Table 1, Table 2, Table 3, Table 4, Table 5 and Table 6, except for the scores. All questionnaires were applied in Japanese. No imputation of missing data was performed. 

Factor analyses were applied to assess the validity of the questionnaire. As an exploratory factor analysis, we performed principal factor analysis with Varimax rotation. The number of components to retain was determined to be five using Kaiser’s criterion (eigenvalue > 1.0), and we confirmed all factor loadings were 0.4 or higher [14]. Following principal factor analysis, confirmatory factor analysis was performed to examine the valid factor structure. The comparative fit index (CFI), adjusted GFI (AGFI), and root mean square error of approximation (RMSEA) were used as indices of conformity. Generally, CFI and AGFI values of ≥0.90 indicate good fits [15]. The RMSEA was also applied (limit for acceptable fit: below 0.06) [16]. Confirmatory factor analysis showed the goodness-of-fit of the five-factor structure models in the questionnaires as follows: CFI = 0.922, AGFI = 0.915, and RMSEA = 0.058.

Cronbach’s alpha interitem consistency coefficient was calculated to test the consistency between statements using a 5-point Likert scale to determine the reliability of these indices. The reliability of all indices was acceptable (alphas of 0.7–0.8). 

**Table 1:** Participant characteristics.

	4th (%)	5th (%)	6th (%)	Clinical Trainee (%)	Total (%)
Responses (%)	89/91 (97.8)	97/100 (97.0)	61/86 (70.9)	39/58 (67.2)	286/335 (85.4)
Sex					
Female	39 (43.8)	48 (49.5)	33 (54.1)	16 (41.0)	136 (47.6)
Male	50 (56.2)	49 (50.5)	28 (45.9)	23 (59.0)	150 (52.4)
Age					
Mean ± SD	23.00 ± 2.07	23.78 ± 1.46	24.90 ± 2.26	25.51 ± 0.94	24.01 ± 2.01
<24	63 (70.8)	43 (44.3)	3 (4.9)	0 (0)	109 (38.1)
24–26	20 (22.5)	47 (48.5)	55 (90.2)	30 (76.9)	152 (53.1)
≥27	6 (6.7)	7 (7.2)	3 (4.9)	9 (23.1)	25 (8.7)
Has silver diamine fluoride (SDF) been included in your didactic classes?					
Yes	74 (83.1)	90 (92.8)	60 (98.4)	37 (94.9)	261 (91.3)
No	15 (16.9)	7 (7.2)	1 (1.6)	2 (5.1)	25 (8.7)
Have you ever used SDF in your clinical practice?					
Yes	0 (0.0)	16 (16.5)	28 (45.9)	18 (46.2)	62 (21.7)
No	89 (100.0)	81 (83.5)	33 (54.1)	21 (53.8)	224(78.3)

SD standard deviation.

**Table 2:** Self-perceived general SDF knowledge.

			Have You Ever Used SDF in Your Clinical Practice?			
	Answers (Score)	Reference	Yes (%)	No (%)	*χ* ^2^	*p* Value
SDF is used for treatment of tooth hypersensitivity.	Yes (1)	[4,17]	31 (50.0)	92 (41.1)		
	No (0)		6 (9.7)	22 (9.8)		
	Don’t know (0)		25 (40.3)	110 (49.1)	1.695	0.428 ^a^
SDF is used to inhibit caries progression.	Yes (1)	[2,17]	58 (93.5)	142 (63.4)		
	No (0)		0 (0.0)	17 (7.6)		
	Don’t know (0)		4 (6.5)	65 (29.0)	21.270	<0.001 ^a^
SDF has anti-microbial properties.	Yes (1)	[2,4]	42 (67.7)	107 (47.8)		
	No (0)		2 (3.2)	14 (6.3)		
	Don’t know (0)		18 (29.0)	103 (46.0)	7.810	0.020 ^a^
SDF has the effect of strengthening the teeth quality.	Yes (1)	[2,17]	27 (43.5)	67 (29.9)		
	No (0)		11 (17.7)	40 (17.9)		
	Don’t know (0)		24 (38.7)	117 (52.2)	4.594	0.103 ^a^
Do you know regarding: How SDF is used to treat dental caries in patients.	Yes (1)		42 (67.7)	43(19.2)		
	No (0)		20 (32.3)	181 (80.8)	–	<0.001 ^b^
Do you know regarding: The advantages SDF treatment can have over traditional treatment.	Yes (1)		25 (40.3)	53 (23.7)		
	No (0)		37 (59.7)	171 (76.3)	–	0.015 ^b^
SDF stains affected (carious) dentin black.	Yes (1)	[4,5,7,17]	44 (71.0)	98 (43.8)		
	No (0)		4 (6.5)	22 (9.8)		
	Don’t know (0)		14 (22.6)	104 (46.4)	14.545	0.001 ^a^
SDF stains unaffected (healthy) dentin black.	Yes (0)	[4,5,7,17]	35 (56.5)	108 (48.2)		
	No (1)		18 (29.0)	21 (9.4)		
	Don’t know (0)		9 (14.5)	95 (42.4)	24.810	<0.001 ^a^
SDF stains skin and clothes.	Yes (1)	[4,5]	58 (93.5)	142 (63.4)		
	No (0)		1 (1.6)	7 (3.1)		
	Don’t know (0)		3 (4.8)	75 (33.5)	21.320	<0.001 ^a^
When applied to deep caries lesions close to the pulp, SDF can cause tooth sensitivity/pain.	Yes (1)	[18]	18 (29.0)	51 (22.8)		
	No (0)		10 (16.1)	22 (9.8)		
	Don’t know (0)		34 (58.4)	151 (67.4)	3.703	0.157 ^a^
Self-perceived general SDF knowledge score						
Mean ± SD			5.85 ±1.98	3.64 ± 2.42	–	<0.001 ^c^

SDF: Silver diamine fluoride, SD: standard deviation. ^a^ Chi-square test, ^b^ Fisher’s exact test, ^c^ Two-tailed *t* test.

**Table 3:** Attitudes on general indications of SDF.

			Have You Ever Used SDF in Your Clinical Practice?			
SDF Treatment Is a Good Treatment Alternative	Answers (Score)	Reference	Yes (%)	No (%)	*χ* ^2^	*p* Value
For restorations in children with behavior issues and dental anxiety.	Strongly agree (2)	[4,5,17]	18 (29.0)	24 (10.7)		
	Agree (1)		31 (50.0)	72 (32.1)		
	Neutral (0)		7 (11.3)	81 (36.2)		
	Disagree (−1)		6 (9.7)	46 (20.5)		
	Strongly disagree (−2)		0 (0.0)	1 (0.4)	28.582	<0.001 ^a^
For medically compromised child.	Strongly agree (2)	[4,5,17]	14 (22.6)	19 (8.5)		
	Agree (1)		16 (25.8)	63 (28.1)		
	Neutral (0)		21 (33.9)	98 (43.8)		
	Disagree (−1)		11 (17.7)	44 (19.6)		
	Strongly disagree (−2)		0 (0.0)	0 (0.0)	9.690	0.021 ^a^
When patients would have to be treated under general anesthesia for their dental treatment.	Strongly agree (2)	[4,5,17]	20 (32.3)	26 (11.6)		
	Agree (1)		12 (19.4)	54 (24.1)		
	Neutral (0)		16 (25.8)	103 (46.0)		
	Disagree (−1)		14 (22.6)	40 (17.9)		
	Strongly disagree (−2)		0 (0.0)	1 (0.4)	18.952	0.001 ^a^
Attitudes score on general indications to use SDF						
Mean ± SD			2.13 ± 2.61	0.86 ± 2.30	–	< 0.001 ^b^

SDF: Silver diamine fluoride, SD: standard deviation. ^a^ Chi-square test, ^b^ Two-tailed *t* test.

**Table 4:** Recognition on general indications of SDF.

			Have You Ever Used SDF in Your Clinical Practice?			
How Much Do You Disagree/Agree with the Following Statements	Answer (Score)	Reference	Yes (%)	No (%)	*χ* ^2^	*p* Value
SDF can be used to arrest the non cavitated lesion in enamel.	Strongly agree (−2)	[4,5,17]	5 (8.1)	25 (11.2)		
	Agree (−1)		4 (6.5)	9 (4.0)		
	Neutral (0)		13 (21.0)	88 (39.3)		
	Disagree (1)		38 (61.3)	99 (44.2)		
	Strongly disagree (2)		2 (3.2)	3 (1.3)	9.641	0.047 ^a^
SDF can be used to arrest the cavitated lesion.	Strongly agree (2)	[4,5,17]	16 (25.8)	18 (8.0)		
	Agree (1)		30 (48.4)	91 (40.6)		
	Neutral (0)		10 (16.1)	95 (42.4)		
	Disagree (−1)		5 (8.1)	20 (8.9)		
	Strongly disagree (−2)		1 (1.6)	0 (0.0)	26.381	<0.001 ^a^
It does not require the use of local anesthesia.	Strongly agree (2)	[4,5,17]	21 (33.9)	37 (16.5)		
	Agree (1)		23 (37.1)	66 (29.5)		
	Neutral (0)		11 (17.7)	89 (39.7)		
	Disagree (−1)		6 (9.7)	31 (13.8)		
	Strongly disagree (−2)		1 (1.6)	1 (0.4)	16.430	0.002 ^a^
The carious dentin must be removed, before applying SDF.	Strongly agree (−2)	[4,5,17]	7 (11.3)	25 (11.2)		
	Agree (−1)		1 (1.6)	5 (2.2)		
	Neutral (0)		21 (33.9)	101 (45.1)		
	Disagree (1)		33 (53.2)	90 (40.2)		
	Strongly disagree (2)		0 (0.0)	3 (1.3)	4.275	0.370 ^a^
It is an alternative to removing tooth structure by a dental drill in order to place restorative material.	Strongly agree (2)	[4,5,17]	7 (11.3)	24 (10.7)		
	Agree (1)		27 (43.5)	56 (25.0)		
	Neutral (0)		20 (32.3)	112 (50.0)		
	Disagree (−1)		7 (11.3)	31 (13.8)		
	Strongly disagree (−2)		1 (1.6)	1 (0.4)	10.266	0.036 ^a^
Knowledge score on practice of SDF						
Mean ± SD			3.06 ± 3.23	1.66 ± 3.04	–	0.002 ^b^

SDF: Silver diamine fluoride, SD: standard deviation. ^a^ Chi-square test, ^b^ Two-tailed *t* test.

**Table 5:** Esthetic acceptability of SDF treatment among dental students.

		Have You Ever Used SDF in Your Clinical Practice?			
SDF Is a Good Treatment to Be Used to Treat Lesions That:	Answer (Score)	Yes (%)	No (%)	*χ* ^2^	*p* Value
Are in the anterior region in primary teeth.	Strongly agree (2)	5 (8.1)	22 (9.8)		
	Agree (1)	24 (38.7)	62 (27.7)		
	Neutral (0)	15 (24.2)	90 (40.2)		
	Disagree (−1)	18 (29.0)	49 (21.9)		
	Strongly disagree (−2)	0 (0.0)	1 (0.4)	6.842	0.144 ^a^
Are in the posterior region in primary teeth.	Strongly agree (2)	11 (17.7)	26 (11.6)		
	Agree (1)	29 (46.8)	67 (29.9)		
	Neutral (0)	12 (19.4)	99 (44.2)		
	Disagree (−1)	10 (16.1)	32 (14.3)		
	Strongly disagree (−2)	0 (0.0)	0 (0.0)	13.360	0.004 ^a^
Are in the anterior region in the permanent teeth.	Strongly agree (2)	1 (1.6)	13 (5.8)		
	Agree (1)	2 (3.2)	23 (10.3)		
	Neutral (0)	10 (16.1)	71 (31.7)		
	Disagree (−1)	48 (77.4)	114 (50.9)		
	Strongly disagree (−2)	1 (1.6)	3 (1.3)	14.710	0.005 ^a^
Are in the posterior region in the permanent teeth.	Strongly agree (2)	2 (3.2)	9 (4.0)		
	Agree (1)	4 (6.5)	34 (15.2)		
	Neutral (0)	10 (16.1)	72 (32.1)		
	Disagree (−1)	45 (72.6)	107 (47.8)		
	Strongly disagree (−2)	1 (1.6)	2 (0.9)	13.071	0.011 ^a^
Esthetic acceptability score on SDF use					
Mean ± SD		−0.45 ± 2.24	0.05 ± 2.66	–	0.172 ^b^

SDF: Silver diamine fluoride, SD: standard deviation. ^a^ Chi-square test, ^b^ Two-tailed *t* test.

**Table 6:** Potential barriers to the use of SDF.

		Have You Ever Used SDF in Your Clinical Practice?			
I Am Not Using/May Not Use SDF Because:	Answer (Score)	Yes (%)	No (%)	*χ* ^2^	*p* Value
I don’t have enough knowledge.	Agree (1)	44 (71.0)	175 (78.1)		
	Disagree (0)	18(29.0)	49 (21.9)	–	0.157 ^a^
Aesthetic is poor.	Agree (1)	44 (71.0)	166 (74.1)		
	Disagree (0)	18 (29.0)	58 (25.9)	–	0.365 ^a^
Patient satisfaction is less.	Agree (1)	31 (50.0)	140 (62.5)		
	Disagree (0)	31 (50.0)	84 (37.5)	–	0.081 ^a^
SDF does not have enough evidence for use.	Agree (1)	15 (24.2)	98 (43.8)		
	Disagree (0)	47 (75.8)	126 (56.3)	–	0.004 ^a^
The potential permanent staining to clothes and counter tops/floors if spilled	Agree (1)	27 (43.5)	138 (61.6)		
	Disagree (0)	35 (56.5)	86 (38.4)	–	0.008 ^a^
Potential barriers score on SDF use					
Mean ± SD		2.60 ±1.67	3.20 ± 1.74	–	0.016 ^b^

SDF: Silver diamine fluoride, SD: standard deviation. ^a^ Fisher’s exact test. ^b^ Two-tailed *t* test.

### 2.3. Statistical Analyses

The data for this survey was entered into an Excel spreadsheet and exported to IBM SPSS Statistics 23.0 and AMOS 23. (Statistical Package for the Social Sciences; SPSS, Chicago, IL, USA). The Shapiro–Wilk test was used to check the normality of the data. Fisher’s exact test and the chi-square test were used to compare categorical variables between students with and without clinical experience with SDF. The two-tailed *t*-test or Kruskal–Wallis test was used to compare the means of continuous variables. Furthermore, a post-test was performed to verify the power of the sample with the program package G*Power software (Power for windows version 3.1.9.4, available from the Heinrich-Heine-Universität Düsseldorf’s website). This indicated that a linear regression model requires a power of at least 0.9 to detect a medium effect size. The significance level was determined to be 0.05 for all statistical tests.

## 3. Results 

Table 1 summarizes the demographic characteristics of the 286 students and clinical trainees (136 women and 150 men) who completed the survey. The mean response rate of all respondents was 85.4%. Among all respondents, 91.3% had been educated about SDF in classroom settings. Among all respondents, 21.7% had experience with SDF in their clinical practice.

Table 2 shows the results regarding general knowledge about SDF, assessed based on the responses to 10 questions. The mean score was significantly higher among the respondents with experience in SDF use than among the respondents with no experience in SDF use (*p* < 0.001). The question with the lowest number of correct answers was “SDF stains unaffected (healthy) dentin black”. The mean score of the sixth-year students was significantly higher than those of the fourth-year and fifth-year students (both *p* < 0.05) (Table 7).

Table 3 represents the attitudes toward the general indications of SDF. The mean score was significantly higher among the respondents with experience in SDF use than among the respondents with no experience in SDF use (*p* < 0.001). The mean score of the sixth-year students was significantly higher than the mean scores of the fourth-year and fifth-year students (both *p* < 0.05) (Table 7).

Table 4 presents the knowledge on the specific indications and practices of SDF. The mean score was significantly higher among the respondents with experience in SDF use than among the respondents with no experience in SDF use (*p* = 0.002).

Table 5 presents the esthetic acceptability of the use of SDF. The respondents experienced with the use of SDF were more likely to agree to treating primary teeth in the posterior region with SDF (*p* = 0.004). The respondents experienced with the use of SDF were more likely to disagree to treating permanent teeth with SDF regardless of the region (*p* = 0.005 and *p* = 0.011, respectively). The mean scores of the sixth-year students and clinical trainees were significantly lower than the mean score of the fourth-year students (both *p* < 0.05) (Table 7).

Table 6 presents the reasons why the respondents do not use/may not use SDF. The mean score was significantly lower among the respondents with experience in SDF use than among the respondents with no experience in SDF use (*p* = 0.016). Among the reasons why the respondents do not use/may not use SDF, the most common reason was not having enough knowledge, followed by poor esthetics. 

## 4. Discussion

In this survey of dental students and clinical trainee dentists in Japan, we evaluated the differences in the knowledge, attitudes, and practices regarding SDF between two groups differentiated by whether they had experience in SDF use. Additionally, we compared the mean scores on five indexes regarding the SDF knowledge and attitudes among the three dental classes and clinical trainees. As the results display, the scores of SDF knowledge and attitude on the general indications for the SDF use increased as the grade level increased. However, significant differences were not found in those scores between sixth-year students and clinical trainees. This might be because clinical trainees had little opportunity to obtain new information about SDF or use SDF on patients after graduating from university.

This survey showed that 91.3% of respondents had been educated about SDF in classroom lectures. A study among pediatric dentists in the United States regarding SDF educational experiences found that 91% of respondents reported that they were not at all educated about SDF in classroom settings and that 95% were not educated about SDF in clinical settings in dental school, this was deemed to be because only one respondent had graduated from dental school in 2015 [9]. A survey among general dental practitioners and pediatric dentists in the Netherlands reported that 22% of respondents had been educated about SDF in basic or post-graduate courses and that knowledge about SDF among dental practitioners was low for the same reasons as in the study in the United States [8]. In contrast, a survey of the knowledge and perceptions of graduating dental students in seven dental schools across the United States regarding SDF reported that almost all of the students recalled receiving information on SDF in the classroom, but many never had the opportunity to apply that knowledge in clinical settings, with 45.2% reporting that they never had used SDF with a patient [13]. These results seem to also apply to Japanese dental students and clinical trainee dentists. Regarding the general knowledge of SDF, for the question “SDF stains unaffected (healthy) dentin black”, more than 70% of respondents answered incorrectly in this survey. The correct answer rate for this question is lower than that in a previous study [13]. Additionally, regarding the question “When applied to deep caries lesions close to the pulp, SDF can cause tooth sensitivity/pain”, over 70% of respondents also answered incorrectly. The correct answer rate for this question is similar to that in a previous study [13]. An in vitro study reported that SDF is cytotoxic to fibroblasts 9 weeks after it is applied to hydroxyapatite discs [18]. This study indicated increased pulp cell death when the remaining dentin thickness between the applied SDF and the pulp was reduced. Although this side effect is mentioned in the document attached to the 38% SDF solution (Saforide^®^, Toyo Seiyaku Kasei Co., Ltd., Osaka, Japan), it may have been difficult for the respondents in this survey to answer correctly without actually encountering this situation. The mean score of knowledge on the practice of SDF in the respondents with no experience in the use of SDF was 1.66 points out of 12 points, and, among the respondents who had experience in using SDF, their mean score was 3.06 points out of 12 points; these findings revealed that the level of our students’ and clinical trainees’ knowledge on the specific indications and practices of SDF is low. Regarding the results related to knowledge and clinical experience, the findings suggest that undergraduate and post-graduate programs do not play a major role in providing knowledge about SDF. 

Studies in countries other than Japan reported that, regardless of their knowledge levels, attitudes toward SDF among dental practitioners were positive [8,9,19]. In this survey, over 50% of respondents with experience in SDF use agreed/strongly agreed that SDF was a good treatment alternative for restorations in children with behavioral issues and dental anxiety, in patients who were medically fragile, and in patients who require general anesthesia for dental treatment. Previous studies on pediatric dentistry program administrators found a high agreement with statements asserting that SDF is indicated for treating patients with behavioral issues and medically compromised patients [9,20]. Another study suggested that the biannual application of 38% SDF for advanced cavitated lesions may be relevant if access to care is limited for uncooperative patients or for patients for whom general anesthesia is not considered safe [21]. However, this survey showed that Japanese dental students and clinical trainees who had no experience using SDF were not necessarily enthusiastic about using SDF. In the case of Japan, the course of SDF use has been different from that in Western countries; SDF was commonly used in the 1970s, but the frequency of SDF use has been decreasing as the incidence of dental caries in children has decreased. We believe that caries treatments for pediatric patients today have become more diverse than in the 1970s, and patients and patients’ families are demanding that dentists select an appropriate method from among several methods. Therefore, it is necessary to review the education program regarding SDF in Japan. 

Among the reasons why the respondents do not use/may not use SDF, the most common reason was their lack of knowledge, followed by poor esthetics. The barriers related to SDF use in this survey are consistent with those reported by previous studies in the United States and Europe [8,9]. Generally, it can be assumed that acquiring more information about SDF is sufficient to increase the use of SDF. For example, this includes increasing the frequency and enriching the content of education programs about SDF. However, it has long been accepted that changing clinical behavior requires more than knowledge; motivation and opportunity are also required [22]. Because the respondents to this survey were dental students or clinical trainees in their first year after graduation, many had never encountered a situation in which they had to spontaneously plan using SDF in clinical practice. As they increase their clinical experience, we expect that there will be more opportunities for them to use SDF spontaneously. 

Among the five indicators, the only one for which no significant difference was observed between the two groups, differentiated by whether they had experience in SDF use, was the “Esthetic acceptability score on SDF use”. The scores of this index became lower as the grade level increased. In other words, as clinical experience increased, the acceptance of using SDF decreased. The American Dental Association (ADA) also demonstrated that permanent staining is observed in arrested caries lesions, limiting its use in esthetic areas [17]. In this survey, more than 70% of respondents who had experience with SDF felt that the use of SDF on permanent teeth was esthetically unacceptable. Another study suggested that the restoration of arrested caries lesions may be needed to recover the form and function of a cavitated tooth, which would also diminish tooth discoloration [23]. It has been reported that the silver-modified atraumatic restorative treatment (SMART), considered a modified application of the atraumatic restorative treatment (ART) philosophy, allows dentists the flexibility to use SDF [24]. In this review, a specific example of this method was described as follows: apply SDF once or more depending on the activity and size of the lesion(s), wait 2 to 4 weeks, and then restore or seal the lesion with the material of choice. Dramatically less or even no caries removal is necessary depending on the hardening or arrest of the lesion [25]. Stains in areas that may show can be selectively excavated (external walls) or blocked with opaquer (internal walls) prior to restoration [24]. We believe that, as a specific strategy for esthetic and morphological restoration, we should first perform restoration using glass ionomer cement (GIC). Previous studies have reported that SDF treatment does not seem to impair GIC bonding [26,27]. If the patient’s cooperation becomes better and a dental drill can be used to restore their teeth, we can perform the sandwich technique using GIC and composite resin [28,29]. 

A recent systematic review reported that the parental acceptance of SDF was better for posterior teeth than for anterior teeth and also for anterior teeth in uncooperative children. Additionally, the parents’ acceptance rate for SDF application increased after follow-up visits and education [30]. It has been suggested that if a child’s parents understand the indications for SDF they will be less reluctant to allow its use. However, this review did not include the results of Japanese surveys, so we need to conduct a survey in Japan to determine the differences in attitudes toward SDF use between parents and dentists.

This study has several limitations, including those described above. Since this survey was conducted within a single university, the actual educational status of students regarding SDF at other universities in Japan is unknown. Therefore, the survey should be expanded to include all university dental schools in Japan, and detailed information about their education should also be collected. In addition, the subjects of this survey were limited to undergraduate students and clinical trainees. It is expected that conducting this study with dentists who have used SDF many times in their practice would likely show more favorable results. In the future, it is necessary to include them in the survey obtain clearer results.

## 5. Conclusions

In the respondents with no experience using SDF, the knowledge level of SDF was lower, and their attitude toward its use was more passive than that of the respondents with experience using SDF. Among the reasons why the respondents do not use/may not use SDF, the most common reason was not having enough knowledge, followed by poor esthetics. The results indicate that dental students and clinical trainees need to increase their clinical experience with SDF use and have more opportunities to encounter cases in which SDF should be used. Consequently, the development and improvement of clinical training programs on SDF for Japanese dental students and clinical trainees are strongly recommended, as well as education on the use of SDF for the dentists who supervise them. Furthermore, it is necessary to identify treatment methods that can reduce the esthetic disadvantages caused by the use of SDF as much as possible.

## Figures and Tables

**Table 7:** Comparisons of the five scores prepared by combining questions for each academic year.

	Academic Year			
	4th (*n* = 89)	5th (*n* = 97)	6th (*n* = 61)	Clinical Trainee (*n* = 39)
Self-perceived general SDF knowledge score	2.65 ± 2.56	4.03 ± 2.11 ^a^	5.66 ± 1.61 ^ab^	5.41 ± 2.31 ^ab^
Attitudes score on general indications to use SDF	0.79 ± 2.15	0.80 ± 2.46	2.03 ± 2.63 ^ab^	1.36 ± 2.25
Knowledge score on practice of SDF	1.37 ± 2.76	1.75 ± 3.29	2.72 ± 3.23	2.67 ± 3.11
Esthetic acceptability score on SDF use	0.74 ± 2.95	0.13 ± 2.59	−0.92 ± 1.84 ^a^	−1.00 ± 1.92 ^a^
Potential barriers score on SDF use	3.03 ± 1.93	3.08 ± 1.78	2.93 ± 1.46	3.33 ± 1.66

SDF: Silver diamine fluoride. Data are expressed as means ± standard deviation. ^a^ *p* < 0.05 vs. 4th-year students (Kruskal-Wallis test). ^b^ *p* < 0.05 vs. 5th-year students (Kruskal-Wallis test).

## Data Availability

The datasets used and/or analyzed during the current study are available from the corresponding author upon reasonable request.
